# Forward genetic screen for auxin-deficient mutants by cytokinin

**DOI:** 10.1038/srep11923

**Published:** 2015-07-06

**Authors:** Lei Wu, Pan Luo, Dong-Wei Di, Li Wang, Ming Wang, Cheng-Kai Lu, Shao-Dong Wei, Li Zhang, Tian-Zi Zhang, Petra Amakorová, Miroslav Strnad, Ondřej Novák, Guang-Qin Guo

**Affiliations:** 1 Institute of Cell Biology and MOE Key Laboratory of Cell Activities and Stress Adaptations, Lanzhou University, Lanzhou 730000, China; 2 Laboratory of Growth Regulators, Centre of the Region Haná for Biotechnological and Agricultural Research, Institute of Experimental Botany ASCR and Palacký University, Šlechtitelů 11, Olomouc CZ-783 71, Czech Republic

## Abstract

Identification of mutants with impairments in auxin biosynthesis and dynamics by forward genetic screening is hindered by the complexity, redundancy and necessity of the pathways involved. Furthermore, although a few auxin-deficient mutants have been recently identified by screening for altered responses to shade, ethylene, N-1-naphthylphthalamic acid (NPA) or cytokinin (CK), there is still a lack of robust markers for systematically isolating such mutants. We hypothesized that a potentially suitable phenotypic marker is root curling induced by CK, as observed in the auxin biosynthesis mutant *CK-induced root curling 1 / tryptophan aminotransferase of Arabidopsis 1* (*ckrc1/taa1*). Phenotypic observations, genetic analyses and biochemical complementation tests of Arabidopsis seedlings displaying the trait in large-scale genetic screens showed that it can facilitate isolation of mutants with perturbations in auxin biosynthesis, transport and signaling. However, unlike transport/signaling mutants, the curled (or wavy) root phenotypes of auxin-deficient mutants were significantly induced by CKs and could be rescued by exogenous auxins. Mutants allelic to several known auxin biosynthesis mutants were re-isolated, but several new classes of auxin-deficient mutants were also isolated. The findings show that CK-induced root curling provides an effective marker for discovering genes involved in auxin biosynthesis or homeostasis.

Auxin plays key roles in the regulation of plant growth and development. The predominant natural form is indole-3-acetic acid (IAA). However, various other small organic acids, both natural (e.g. 4Cl-IAA and phenylacetic acid) and synthetic (e.g. 2,4-dichlorophenoxyacetic acid and 1-naphthaleneacetic acid; 2,4-D and NAA, respectively) also exhibit auxin-like activities^1^.

In plants, IAA is produced by multiple highly connected biosynthetic pathways^2^,^3^. It is then transported between cells by various efflux and influx carrier proteins that create so-called auxin maxima and concentration gradients, which play important regulatory roles in embryogenesis and organogenesis^4^. *In planta*, auxin signaling is initiated by auxin binding to receptors such as TRANSPORT INHIBITOR RESPONSE1/AUXIN SIGNALING F-BOX (TIR1/AFB) receptors^5^, AUXIN BINDING PROTEIN 1 (ABP1)^6^,^7^, and the Arabidopsis cell cycle F-box protein S-phase kinase-associated protein 2A (SKP2A)^8^. The best characterized TIR1/AFB-Aux/IAA co-receptor system regulates auxin-dependent transcription within the nucleus, and auxin binding enables SKP2A to mediate degradation of transcription factors that repress cell division, such as E2-promoter binding factor C (E2FC) and E2F dimerization partner B (DPB)^8^. Despite the obvious auxin binding activity of ABP1 and its reported involvement in the so-called fast, nontranscriptional cellular responses and some developmental processes^6^,^7^,^9^, recent work with *abp1* null mutants suggests that ABP1 is not essential for Arabidopsis development under normal conditions^10^.

Auxin biosynthesis is less well understood than auxin transport and signaling, but includes several tryptophan (Trp)-dependent and -independent pathways^11^. Four Trp-dependent pathways have been proposed, named according to the major putative intermediates: the indole-3-pyruvic acid (IPA), indole-3-acetamide (IAM), tryptamine (TAM) and indole-3-acetaldoxime (IAOx) pathways^3^. Only the IPA pathway has been completely characterized at genetic and biochemical levels to date. It involves two reactions that convert L-Trp to IAA, sequentially catalyzed by TRYPTOPHAN AMINOTRANSFERASE OF ARABIDOPSIS 1 (TAA1) and YUCCA (YUC) flavin monooxygenases^12^,^13^,^14^. The genetic bases, potential intermediates and significance for IAA biosynthesis (at least in Arabidopsis) of the Trp-independent pathway(s) are all uncertain^15^.

The multiple interconnected biosynthesis pathways and their high genetic redundancy complicate efforts to detect auxin-deficient mutants (which would facilitate dissection of the pathways) via direct genetic screening. A further hindrance is the lack of a reliable phenotypic marker for screening auxin-deficient mutants. Some genes involved in auxin biosynthesis have been identified by isolating mutants with high auxin contents^16^,^17^,^18^,^19^ or characterizing mutants isolated in studies that initially focused on indirectly associated phenomena^13^,^14^,^20^,^21^,^22^. However, only a few auxin-deficient mutants have been detected by such indirect screens^2^,^14^, and there is still a lack of a systematic forward genetic screening procedure for isolating auxin-deficient mutants^3^,^23^.

We hypothesized that a potentially useful marker may be root curling induced by cytokinin (CK), as recently described in the auxin biosynthesis mutant *CK-induced root curling 1 / tryptophan aminotransferase of Arabidopsis 1 (ckrc1/taa1*), arising from auxin deficiencies in the root tip^20^. To test the possibility that this trait could be used to isolate more auxin-deficient mutants, we screened large collections of Arabidopsis T-DNA insertion lines, obtained from the Arabidopsis Biological Resource Center (ABRC), to isolate *ckrc*-like mutants. The advantage of *Agrobacterium* T-DNA over classical mutagens is that the plant sequences flanking the insertion site can be isolated easily. This simplifies the identification of genes corresponding to interesting mutants. A number of pools of T-DNA insertion lines are now available with the coverage of the whole genome^24^,^25^. Our results show that the trait can be effectively applied to isolate mutants with perturbations in auxin biosynthesis, transport and signaling. Furthermore, mutants with auxin-deficiencies can be rescued by exogenous auxins. Mutants carrying mutations in several previously identified biosynthesis genes were re-isolated, and some previously unknown auxin-deficient mutants with significantly reduced endogenous auxin levels were also isolated. The results clearly suggest that the screening system is effective for isolating auxin-deficient mutants, and hence discovering genes that participate in auxin biosynthesis or dynamics.

## Results

### Forward genetic screen for *ckrc* mutants. 

**Figure 1:**
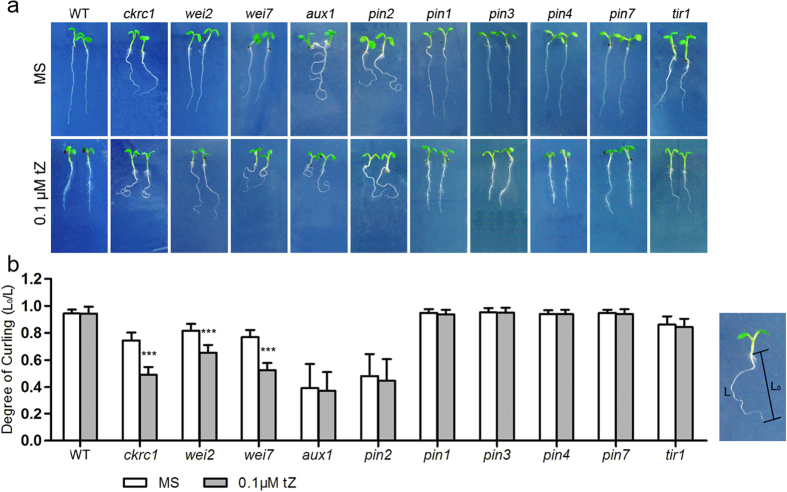
Comparison of 7 DAG root phenotypes between WT and indicated auxin mutants. (**a**) Effect of tZ on indicated mutants. (**b**) The Degree of root Curling (DC) was calculated by dividing the distance between the two ends of seedlings’ roots (L_0_) by the length of their roots (L). DC of roots of WT seedlings and indicated mutants in the presence and absence of 0.1 μM tZ. Presented data are means ± SD (n = 30). Asterisks indicate statistically significant differences between Mock- and 0.1 μM tZ-treated seedlings according to t-tests (*, **, and *** correspond to P-values of 0.05 > p > 0.01, 0.01 > p > 0.001, and p < 0.001, respectively).

To date few auxin-deficient mutants have been directly isolated by forward genetic screening^14^, partly because of a lack of suitable markers. However, we have previously shown that CK up-regulates local auxin biosynthesis in root tips but inhibits polar auxin transport (PAT), and exogenous CKs induce strong root curling in the auxin biosynthesis mutant *ckrc1/taa1*^20^. To test the hypothesis of CK-induced root curling (ckrc) could be used as a marker for detecting other auxin-deficient mutants, we first applied CK to two other Arabidopsis mutants, *weak ethylene insensitive 2* (*wei2*) and *wei7*. These mutants have reduced auxin levels due to mutations in genes encoding α- and β-subunits, respectively, of anthranilate synthase, a rate-limiting L-Trp biosynthetic enzyme^22^. We measured their Degrees of Curling (DC) to compare them with those of WT and *ckrc1* plants. As expected, when grown vertically on Murashige and Skoog (MS) medium in the presence of 0.1 μM *trans*-Zeatin (tZ), their primary roots curled, although the phenotype of *wei2* roots was relatively weak (Fig. 1a,b).

We also tested auxin transport mutants *auxin resistant 1* (*aux1*) and *pin-formed 2* (*pin2*), which have impairments in auxin influx and efflux transport, respectively, and both reportedly have defective root gravitropic responses^26^,^27^,^28^,^29^. In contrast to the auxin-deficient mutants *ckrc1*, *wei2* and *wei7*, the strong curling of roots of *aux1* and *pin2* mutants did not depend on the presence of tZ, thus displaying a constitutive root curling (crc) phenotype (Fig. 1a & Table 1). However, roots of *pin1*, *pin3*, *pin4* and *pin7* auxin efflux mutants showed no obvious curling on either tZ-containing or basal MS medium (Fig. 1a). The auxin signaling mutant *tir1*, defective in an auxin receptor, displayed weak wavy root rather than a curling root phenotype, which was not significantly affected by 0.1 μM tZ (Fig. 1a). Together, these results suggest that the ckrc phenotype differs from those of auxin transport and signaling mutants, and may allow large-scale genetic screens for auxin-deficient mutants, which have not been previously reported^3^.

To optimize screening conditions, *ckrc1*, *wei2* and *wei7* mutant seeds were germinated and grown on MS plates with tZ in a range of concentrations, and then their root phenotypes were scored 7 days after germination (DAG). Root curling was most distinct at 0.1–1 μM tZ, and became weak or masked by growth inhibition at higher tZ concentrations (Supplementary Fig. S1). We chose 0.1 μM tZ as the general screening concentration (for some mutants with weak responses <0.1 μM tZ was better, see below). In its presence, mutants with either curling or wavy root phenotypes were isolated by a two-round screening procedure (putative mutants detected in the first round were confirmed by the second) (Supplementary Fig. S2 & Table 2). In an initial large-scale application of this procedure to two collections of Arabidopsis lines, CS76502/4/6/8 (40,000 lines)^24^ and CS31100 (62,000 lines)^25^, we isolated 53 mutants with curled or wavy roots. A further 96 mutants were subsequently isolated after screening the other nine stocks listed in Table 2. In the following sections we present results of genetic and phenotypic characterizations of the first set of 53 isolated mutants. Similar investigations of the other 96 mutants are ongoing and some significant results obtained from their analyses are also noted below.

**Figure 2:**
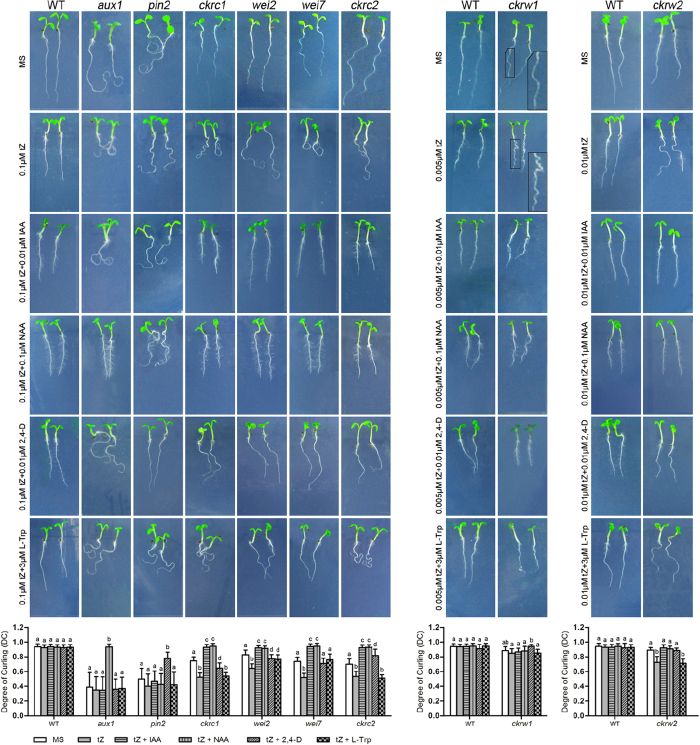
Biochemical complementation tests of the 7 DAG curled/wavy root mutants and WT controls. Seedlings were grown on MS plates with and without indicated combinations of tZ, auxins and L-Trp. Presented data obtained in the DC analysis are means ± SD (n = 30). Different letters indicate significant differences at P < 0.05, according to ANOVA followed by Tukey’s multiple comparison tests.

When grown on MS without tZ, the curling root phenotype of some of the isolated mutants was maintained. These were designated group I, *constitutive root curling* (*crc*), mutants. In most of the others the trait was either significantly weaker or disappeared. These were designated group II, *CK-induced root curling* (*ckrc*), mutants. A third group consisted of two *CK-induced root waving* (*ckrw*) mutants, *ckrw1* and *ckrw2*, whose phenotypes were most distinct at 0.005 and 0.01 μM tZ concentrations, respectively (Fig. 2). On MS without tZ, the ck*rw1* phenotype became weak (displaying a longer ‘wavelength’ with a lower ‘frequency’); while the ck*rw2* phenotype disappeared (Table 1).

Genetic analysis of the progenies produced from backcrosses of these isolated mutants to their respective WTs revealed that WT complemented all of them in the F_1_ generation and showed 1:3 segregation ratios in the F_2_ generation, indicating that they carried mutations in single recessive genes (Supplementary Table S1).

### Biochemical complementation and allelism tests

The curled root phenotype of auxin-deficient mutants can be rescued by exogenous auxins^20^, and both influx and efflux transport mutants display differential sensitivities to different kinds of auxins^30^,^31^. Moreover, L-Trp can rescue auxin-deficient mutants that are defective in L-Trp biosynthesis^22^ (Table 1), but not true auxin-biosynthesis mutants, i.e. mutants with perturbations downstream of L-Trp production^20^. To primarily distinguish between these possibilities, we performed biochemical complementation and genetic allelic tests.

Mutant seedlings were grown on vertical MS/tZ plates with selected auxins or L-Trp, and their root phenotypes were observed. The results showed that the group I *crc* mutants could be divided into two subgroups: one only rescued by NAA and the other only by 2,4-D, which are typical traits of the influx carrier mutant *aux1* and the efflux carrier mutant *pin2*, respectively (Fig. 2 & Table 1). Allelism tests revealed that their mutations are indeed allelic to *aux1* or *pin2* (Table 1 & Supplementary Table S1).

Unlike group I, the group II *ckrc* mutants can be rescued by all three of the auxins used, as previously reported^20^, although often only partly by 2, 4-D. However, they could be divided into two classes by their responses to L-Trp (Table 1). Two currently known auxin-deficient mutants, *wei2* and *wei7*, can be rescued by L-Trp^22^, but not *ckrc1/taa1* (Fig. 2)^13^,^20^. Thus, we performed allelism tests by genetic crossing with these three known mutants. The results showed that some group II mutants are allelic to *ckrc1*, *wei2* or *wei7* mutants (Table 1). However, one group II mutant is not allelic to any of these three known mutants, and was renamed *ckrc2* (Fig. 2 & Table 1). Three such group II mutants were also identified among the 96 subsequently isolated mutants (Supplementary Table S2), and gene cloning confirmed that they carry mutations in genes that have not been identified as mutated in any previously characterized auxin-deficient mutants.

The wavy-root mutants *ckrw1* and *ckrw2* showed different responses in the biochemical complementation tests. While all three auxins could rescue *ckrw2*, like the group II mutants, only 2, 4-D had rescuing effects on *ckrw1* mutants (Fig. 2 & Table 1). Allelism tests showed that these two mutants are not allelic to *taa1*, *wei2*, *wei7*, *aux1* or *pin2*, suggesting that they represent new classes of auxin mutants.

### IAA can stimulate root growth and restore root gravitropic responses of *ckrc2*, *ckrw1* and *ckrw2* at low concentrations

**Figure 3:**
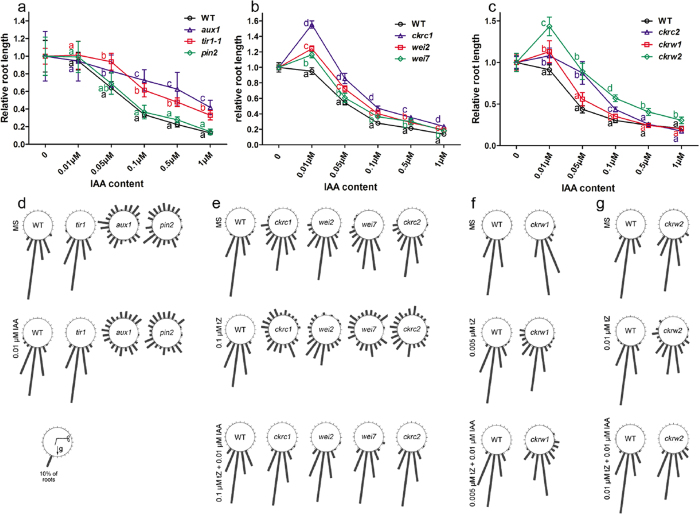
Effects of exogenous IAA on WT seedlings and indicated auxin-mutants. (**a**–**c**) Primary root elongation measured on 7 DAG; means ± SD (n > 20). Different letters indicate significant differences at P < 0.05 according to ANOVA followed by Tukey’s multiple comparison tests. (**d**–**g**) Exogenous IAA can restore root gravitropism in *ckrc* or *ckrw* mutants. Approximately 80 seedlings were assessed per genotype or treatment.

As shown above, exogenous auxins rescued phenotypes of *ckrc2* and *ckrw2* mutants in the biochemical complementation tests (Table 1), corroborating their putative auxin-deficiency. Roots of auxin-deficient mutants usually display positive growth responses to exogenous IAA at low concentrations, in contrast to negative responses of WT plants, and weak gravitropic responses^20^. In further tests reported here, we found that the *aux1* auxin influx carrier mutant and *tir1* auxin signaling mutant were significantly less sensitive than WT to IAA in a root growth inhibition assay, while responses of the *pin2* auxin efflux carrier mutant were more similar to WT (Fig. 3a). Like the *ckrc1* mutant^20^, at low concentration IAA stimulates rather than inhibits the root growth of *ckrc2*, *ckrw1* and *ckrw2* mutants (Fig. 3b,c). Accordingly, 0.01 μM IAA also rescued the defective or weak gravitropic responses of *ckrc2* and *ckrw2* mutants (Fig. 3e,g). In contrast, exogenous IAA did not restore gravitropism in *aux1* and *pin2* mutants (Fig. 3d). Paradoxically, 0.01 μM IAA stimulated *ckrw1* root growth (Fig. 3c) and partially rescued its gravitropic response (Fig. 3f), but had little effect on its wavy-root phenotype (Fig. 2).

### *ckrc2*, *ckrw1* and *ckrw2* respond normally to IAA and tZ

**Figure 4:**
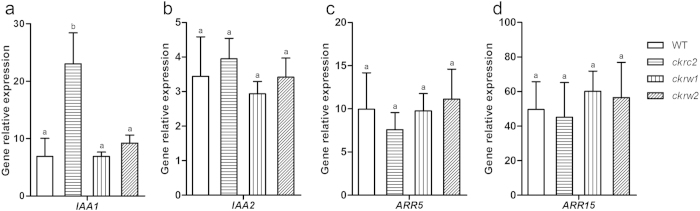
Induction of expression of auxin/CK primary response genes by exogenous IAA (**a**, **b**) or tZ (**c**, **d**) treatments in WT and indicated mutants. Transcript levels in treated seedlings relative to untreated controls, normalized to *ACT-8* mRNA levels. Presented data are means ± SD (n = 4). Different letters indicate significant differences at P < 0.05 according to ANOVA followed by Tukey’s multiple comparison tests.

To see if auxin or CK signaling was affected in *ckrc2*, *ckrw1* and *ckrw2* mutants, we analyzed their expression of two auxin (*IAA1/2*) and two CK (*ARR5/15*) primary response genes after treatment with these hormones by real-time quantitative PCR (qRT-PCR). As shown in Fig. 4, although the gene response strengths differed among them, none of the mutants showed significantly weaker responses than WT plants, implying that they were not defective in auxin or CK signaling.

### Endogenous auxin contents are low in *ckrc* and *ckrw* mutants

**Figure 5:**
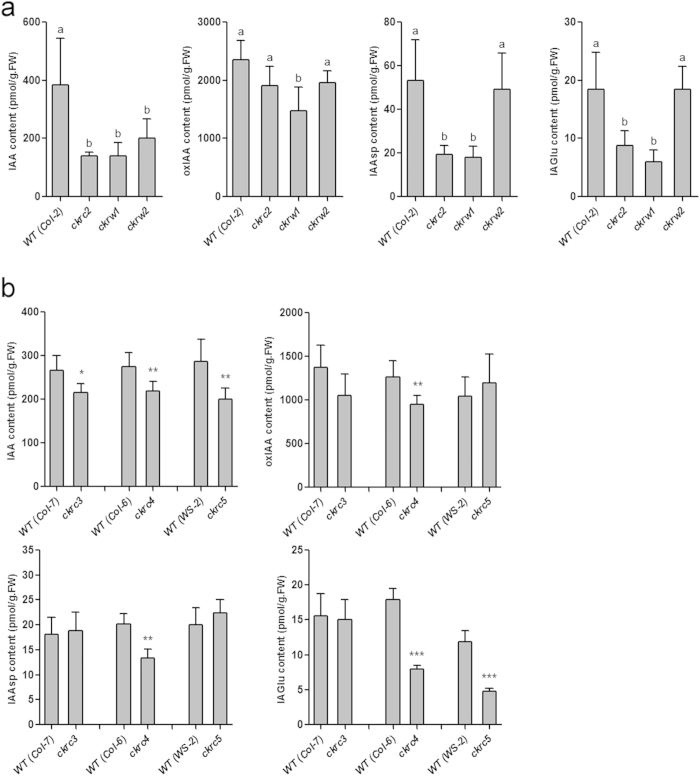
Endogenous levels of IAA and its metabolites. The data were measured in 7-d-old mutant and respective WT seedlings; means ± SD (n = 4). (**a**) Different letters indicate significant differences at P < 0.05 according to ANOVA followed by Tukey’s multiple comparison tests. (**b**) Asterisks indicate statistically significant differences between the mutants and WT according to t-tests (*, **, and *** correspond to P-values of 0.05 > p > 0.01, 0.01 > p > 001, and p < 0.001, respectively). oxIAA, 2-oxindole-3-acetic acid; IAGlu, IAA-glutamate; IAAsp, IAA-aspartate.

As three tested mutants showed traits associated with auxin-deficiency, such as weak gravitropic responses and positive root growth responses to IAA at low concentrations, we directly measured their endogenous contents of free IAA and its metabolites by LC-MRM-MS^32^. Like the auxin-biosynthesis mutant *ckrc1/taa1*, significant overall reductions in levels of both free IAA and most of its metabolites (oxIAA, IAGlu and IAAsp) were observed, especially in *ckrc2* mutants (Fig. 5a), suggesting that they are probably true auxin deficient mutants. Reduced levels of free IAA were also detected in the subsequently isolated *ckrc3*, *ckrc4* and *ckrc5* mutants (Fig. 5b).

### Flanking sequences and/or genetic mapping

In order to identify the mutated genes, we used Tail-PCR to amplify the T-DNA flanking sequences or high-throughput sequencing to identify the inserted genes. Overall, 1-5 T-DNA independent insertions were identified per mutant, in accordance with the frequent presence of more than one insertion in T-DNA transformants.

Three T-DNA insertions were identified in *ckrw1*, but only one (in AT5G49665, *WAVY GROWTH 3*, *WAV3*^33^), showed genetic linkage to this mutant, and genetic crossing confirmed that it was allelic to *wav3*.

The T-DNA insertions in *ckrc2* and *ckrc3* were not genetically linked to their curled root phenotypes. However, map-based cloning located the *ckrc*2 mutation in the region between the T5F17 and F16A16 markers in chromosome 4, and the *ckrc3* mutation between MCK7 and MZN1 in chromosome 5. AT4G28720 (*YUC8*^34^) and AT5G58450 (*TRANSCURVATA2*, *TCU2*^35^) genes are located in these regions, and allelism tests confirmed that *ckrc*2 and *ckrc3* are allelic to *yuc8* and *tcu2*, respectively.

In the *ckrc5* mutant, no T-DNA insertion was detected either, but genomic DNA sequencing revealed a deletion in the gene AT3G02260 (*TIR3/BIG*^36^), and allelism tests confirmed that it is allelic to *tir3*/*big*.

## Discussion

We have established an effective genetic screening protocol for isolating auxin-deficient mutants by using CK-induced root curling (ckrc) or root waving (ckrw) as a phenotypic marker. Substantial numbers of mutants have been identified using the protocol. Most are allelic to known mutants with perturbations in auxin transport genes (*aux1* or *pin2* in group I *crc* mutants) or biosynthesis (*taa1/ckrc1* and the two L-Trp biosynthetic mutants *wei2* and *wei7*). However, six (*ckrc2*, *ckrw1*, *ckrw2*, *ckrc3*, *ckrc4* and *ckrc5*) are not allelic to any known auxin-deficient mutants in previous forward genetic screens (Tables 1 & Supplementary Table S2). Notably, all of the isolated mutants that are allelic to known biosynthetic mutants are group II *ckrc* mutants (Tables 1 & Supplementary Table S2). Thus, this group appears to consist of (or is enriched in) auxin-deficient mutants with perturbations in auxin biosynthesis or homeostasis. Notably, no allele of known CK or other hormonal mutants was isolated in our screen, suggesting that the curled/wavy root phenotype is specifically related to mutations affecting auxin-mediated processes. This is similar to *tir* mutations, which reportedly affect auxin signaling (*tir1/5*), transport (*tir3*) or biosynthesis (*tir2/7*)^21^,^37^, but different from *wei* and *sav* mutations, which include perturbations in the ethylene receptor *ERS* (*wei4*), the *EIN3*-related transcription factor gene *EIL1* (*wei5*)^38^, a C-22 hydroxylase involved in brassinosteroid biosynthesis (*sav1*) and a β-tubulin isoform (*sav2*)^14^.

In the reported large-scale genetic screen we isolated auxin-deficient mutants that are not allelic to any previously detected in forward genetic screens. They were not defective in auxin/CK signaling responses (Fig. 4), but displayed some typical auxin-deficiency traits, such as weak gravitropic responses (Fig. 3e–g), positive root growth responses to auxin at low concentrations (Fig. 3c), and reduced levels of endogenous IAA and its metabolites (Fig. 5). This is consistent with recent observations of overall reductions in levels of IAA, oxIAA, IAAsp and IAGlu in auxin-biosynthesis mutants (and increases in auxin over-production mutants)^32^,^39^.

Our genetic mapping and allelism tests showed that *ckrc2* is allelic to *yuc8*. The *YUC* flavin monooxygenases were initially proposed to catalyze conversion of tryptamine to N-hydroxytryptamine, but are now believed to convert IPA to IAA^12^,^40^. Strong functional redundancy has been found among *YUC*s^34^,^41^, and no single *yuc* mutant has been previously detected in forward genetic screens. Thus, the successful isolation of *ckrc2/yuc8* reveals the utility of the ckrc marker for isolating auxin-deficient mutants. Moreover, the CK-associated root phenotypes of *ckrc2* mutants suggest that *YUC8* participates in the mediation of root responses to CK. Hence, *YUC8* may play a more important general signaling role than previously suspected, as recent reverse genetic evidence indicates that it also mediates jasmonic acid (JA) signaling and hypocotyl growth responses to temperature changes^42^,^43^.

The *ckrw1* and *ckrw2* mutants showed phenotypic deviation (wavy rather than curling roots) from the typical group II *ckrc* mutants, but their characteristic traits could still be induced or intensified by CK. Our genetic and molecular analyses indicate that *ckrw1* is an allele of *WAV3*, which encodes a RING-containing protein with E3 ubiquitin ligase activity *in vitro*^33^. The inability of IAA fully rescue *ckrw1* in biochemical complementation tests, despite their reduced endogenous IAA levels (Fig. 5), suggests that this mutant is probably not simply auxin-deficient. Auxin signaling or transport may be also affected in it, as suggested by Sakai *et al.* (2012)^33^.

Identification of *ckrc3* and *ckrc5* as alleles of *tcu2*^35^ and *tir3/big*^36^,^37^, respectively, and their auxin-deficiencies revealed in our screen suggests that these two genes participate in auxin homeostasis. The *TCU2* gene encodes the auxiliary subunit of the NatB N-α-acetyltransferase complex, which is required for N-α-terminal acetylation of proteins in Arabidopsis. Mutation of *TCU2* causes pleiotropic perturbations in leaf, flower and seed development^35^. The auxin-deficiency of the *ckrc3/tcu2* mutant revealed in our study implies that NatB-mediated N-α-terminal acetylation is required for the maintenance of proper auxin levels.

Previous investigations indicate that TIR3/BIG participates in positioning of auxin efflux carriers at the plasma membrane, and thus in polar auxin transport^36^,^37^,^44^. Hence, its mutation may affect diverse hormone and light responses^45^. Its precise role in PAT is still obscure, but our biochemical complementation results and auxin measurements suggest that BIG may also be involved in auxin homeostasis. It should be noted that similar features, such as low DR5-GUS activities in auxin biosynthesis sites at root tips or young cotyledon/leaf margins have also been reported^46^,^47^,^48^. Moreover, auxin itself can affect the cellular location of its carrier proteins^6^,^44^, and reduced basipetal auxin transport has been reported in auxin biosynthesis mutant *ckrc1*^20^.

Similarly to auxin-deficient mutants previously detected in forward genetic screens, the *ckrc/ckrw* mutants we isolated are weak, in the sense that they have reduced auxin levels and their mutations have no severe growth or developmental effects. They were able to complete their life cycle and retained fertility under normal growth conditions. More serious perturbations, such as defects in embryogenesis and radically abnormal development with infertility, have only been reported in double or multiple mutants of auxin biosynthesis genes^13^, clearly suggesting strong functional redundancy.

The characteristic traits of the mutants isolated from the activation-tagged T-DNA lines^25^ in this study were recessive, indicating that they were caused by loss- rather than gain-of-gene-function mutations. The high frequencies of *aux1*/*ckrc1*/*pin2* mutants may be explained by their relatively strong phenotypes (making them easy to identify in screens). Mutants with weaker phenotypes are inevitably more difficult to detect, but may be distinguishable under subtle changes in growth conditions. Thus, with further modifications of the selection conditions (such as variations in CK concentration and/or temperature) and screening more mutants, including sets of chemically/physically induced mutants, we may approach saturated (genomic scale) screening for auxin-deficient mutants.

## Methods

### Plant material and growth conditions

Collections of Arabidopsis thaliana T-DNA insertion lines (Table 2) were purchased from the Arabidopsis Biological Resource Center (ABRC) (http://abrc.osu.edu/), then germinated and cultivated, as previously described^20^, at 25 °C with a 16 h light / 8 h dark cycle. For growth analyses, seedlings were grown on vertical plates of MS medium solidified with 1.0% w/v agar (hereafter MS plates) supplemented with 10 g/L sucrose.

Arabidopsis thaliana accessions Col-0, 2, 6, 7, C24 and Ws-2 were used as WT. Control *wei2* (*wei2-1/DR5::GUS*) (N16397), *wei7* (*wei7-2/DR5::GUS*) (N16436), *aux1* (*aux1-7/DR5::GUS*) (N16704), *pin2* (*eir1-*1/*DR5::GUS*) (N16706), *pin1* (N5220), *pin3* (N9363), *pin4* (N9368), *pin7* (N9365) and *tir1-1* (N3798) mutants were purchased from the Nottingham Arabidopsis Stock Centre (NASC).

### Mutant screening and genetic analysis

For the mutant screening, seeds from the T-DNA insertion lines were surface-sterilized and germinated on MS plates containing 0.1 μM tZ (Sigma, http://www.sigmaaldrich.com). Putative mutant seedlings (M_1_) with curled/wavy roots were identified 10–14 days later. They were planted in soil to harvest seeds (M_2_) for the second round of screening (Supplementary Fig. S2) and the confirmed mutants were subjected to further genetic analysis.

For genetic analysis, mutants were backcrossed to their respective WTs and the F_1_ phenotypes were observed. Genetic segregation ratios were calculated from observations of F_2_ generations.

For genetic allelic tests, the known and newly isolated mutants were reciprocally crossed and the phenotypes of the resulting F_1_ plants were determined.

### Phenotypic characterization

The Degree of root Curling (DC) was calculated by dividing the distance between the two ends of seedlings’ roots (L_0_) by the length of their roots (L). For biochemical complementation assays, mutant seedlings were grown on vertical MS/tZ plates with various auxins or L-Trp (Sangon, http://www.sangon.com), then their root phenotypes were observed.

For root growth inhibition assays, seedlings were germinated and grown on vertical MS plates supplemented with IAA (Sigma) at a selected range of concentrations for 7 days then their root elongation was measured^20^. All presented data for these assays are means obtained from three separate experiments, each with at least 20 seedlings.

For root gravitropic assays, seeds were germinated and grown on vertical MS plates at 25 °C. Five days later, the seedlings were transferred to fresh media containing no hormones, IAA, tZ or both at concentrations indicated in Fig. 3d–g. Three hours later, the plates were rotated through 90° and their root bending angle was measured after further incubation for sufficiently long to generate a measurable bend (>12 h, depending on the root growth rates. Approximately 80 seedlings were assessed per genotype or treatment.) The frequencies (%) of root growth direction at intervals of 15° are represented by the lengths of the bars.

### Gene cloning and sequence analysis

Tail-PCR was used to clone the T-DNA flanking sequences in the isolated mutants^49^. All PCR products were electrophoretically separated in 1% agarose gel, and the expected TAIL-3 products were purified and sequenced. DNA sequences were aligned with blastn (http://www.ncbi.nlm.nih.gov/BLAST/) and Tair10 (http://www.arabidopsis.org/Blast/) programs.

For the putative new mutants (see Results), T-DNA flanking sequences were also detected by high-throughput sequencing (at ShangHai Biotechnology Corporation, China, http://www.shbiotech.org/, and at Hangzhou Guhe Information and Technology Co., Ltd, China, http://www. guheinfo.com/). If no linkage was found between any detected T-DNA inserts and a mutant’s phenotype, map-based cloning was performed using F_2_ populations generated by backcrossing the mutants to their respective WTs, as previously described^50^.

### RNA preparation and expression analysis

Arabidopsis seedlings were immediately frozen in liquid nitrogen, and stored at −80 °C. RNA was isolated using Trizol (Sangon) and reverse-transcribed using a reverse transcription kit (DRR047A) (Takara, http://www.takara-bio.com/). Quantitative RT-PCR was performed using a Bio-Rad CFX96^TM^ Real-time System (Bio-Rad, http://www.bio-rad.com) and Power SYBR green chemistry (DRR081A) (Takara), with primers listed in Supplementary Appendix S1.

CK- and auxin-inducible gene expression was analyzed in 7-day-old seedlings grown on MS medium, as previously described^20^, using treatments consisting of exposure to 10 μM tZ for 30 min^51^ or 20 μM IAA for 1.5 h^52^ in liquid MS medium. There were four independent biological replicates per treatment.

### Endogenous auxin measurement

Endogenous levels of free IAA and its metabolites were measured using previously described LC-MS/MS methods^32^. Briefly, samples (20 mg fresh weight) of 10-day-old Arabidopsis seedlings were collected, extracted in ice-cold 50 mM sodium phosphate buffer (pH 7) and purified by SPE on hydrophilic-lipophilic balanced reversed-phase sorbent columns (Oasis^®^ HLB, 1 cc/30 mg, Waters). To each extract, 5 pmol of ^13^C_6_-IAA, ^13^C_6_-oxIAA, ^13^C_6_-IAAsp and ^13^C_6_-IAGlu were added as internal standards to validate the quantification. All samples were then evaporated at 37 °C to dryness *in vacuo*. Purified samples were analyzed by the LC-MS/MS system consisting of an ACQUITY UPLC^®^ System (Waters) and Xevo™ TQ-S (Waters) triple quadrupole mass spectrometer. Quantification was obtained using a multiple reaction monitoring (MRM) mode of selected precursor ions and the appropriate product ion^32^. The linear range spanned at least five orders of magnitude with a correlation coefficient of 0.9989–0.9998. Four independent biological replicates of each mutant were used in these analyses.

## Additional Information

**How to cite this article**: Wu, L. *et al.* Forward genetic screen for auxin-deficient mutants by cytokinin. *Sci. Rep.* **5**, 11923; doi: 10.1038/srep11923 (2015).

## Supplementary Material

Supplementary Information

## Figures and Tables

**Table 1:** Results of biochemical complementation and allelism tests on the 53 curled/wavy root mutants isolated from the CS76502/4/6/8 and CS31100 stocks.

Group	Subgroup	Phenotype						No. mutants	Allele
		MS	tZ	tZ+IAA	tZ+NAA	tZ+2,4D	tZ+L-Trp		
I (crc)	*aux1*-like	++	+++	++	−	++	+++	20	*aux1*
	*pin2*-like	++	+++	++	++	−	+++	10	*pin2*
II (ckrc)	*taa1*-like	+	+++	−	−	+	+++	13	*taa1*
		+	+++	−	−	+	+++	1	c*krc2*
	*wei2*-like	−	+	−	−	<+	<+	2	*wei2*
	*wei7*-like	−	++	−	−	+	−	5	*wei7*
III (ckrw)	*ckrw1*	+	++	<++	<++	+	++	1	*ckrw1*
	*ckrw2*	−	+	−	−	<+	+	1	*ckrw2*

+++: ‘strong curling’; ++: ‘curling’; +: ‘weak curling’; -: ‘wild-type phenotype’.

**Table 2:** Summary information about the pools screened and the 149 isolated mutants with curled/wavy roots following exposure to tZ.

Pools	Vector	Ecotype	No. of lines	No. of seedlings screened	No. of isolated mutants
CS31100	pSKI015	Col-2	~62,000	~630,000	40
CS76502/4/6/8	pROK2	Col-0	~40,000	~450,000	13
CS 31400	pSKI015	C24	~9,700	~24,700	2
CS 31402	pSKI015	C24	~4,000	~10,320	1
CS 21991	pSKI015	Col-7	~8,200	~108,810	6
CS 21995	pSKI015	Col-7	~8,600	~48,860	1
CS 23153	pSKI015	Col-7	~6,200	~63,900	16
CS 31087	pD991	Col-6	~11,300	~113,060	16
CS 22830	pD991-AP3	Ws-2	~37,800	~408,140	11
CS 6502	3850:1003	Ws-2	~6,500	~71,580	4
CS 84442	3850:1003	Ws-2	~4,000	~40,050	39
Total			~198,300	~1,969,420	149
