# Supplementary material for: Forward genetic screen for auxin-deficient mutants by cytokinin
Source: Sci Rep. 2015 Jul 6;5:11923. doi: 10.1038/srep11923 (PMC4491711; doi:10.1038/srep11923)
Supplement: Supplementary Information [file srep11923-s1.pdf]

Forward genetic screen for auxin-deficient mutants by cytokinin

Lei Wu[1,+], Pan Luo[1,+], Dong-Wei Di[1,+], Li Wang[1], Ming Wang[1], Cheng-Kai Lu[1], Shao-Dong Wei[1], Li Zhang[1], Tian-Zi Zhang[1], Petra AMAKOROVÁ[2], Miroslav STRNAD[2], Ondřej NOVÁK[2] and Guang-Qin Guo[1,*]

[1]Institute of Cell Biology and MOE Key Laboratory of Cell Activities and Stress Adaptations, Lanzhou University, Lanzhou 730000, China

[2]Laboratory of Growth Regulators, Centre of the Region Haná for Biotechnological and Agricultural Research, Institute of Experimental Botany ASCR and Palacký University, Šlechtitelů 11, Olomouc CZ-783 71, Czech Republic

[*]Corresponding. gqguo@lzu.edu.cn

[+]These authors contributed equally to this work

The author(s) declare no competing financial interests.

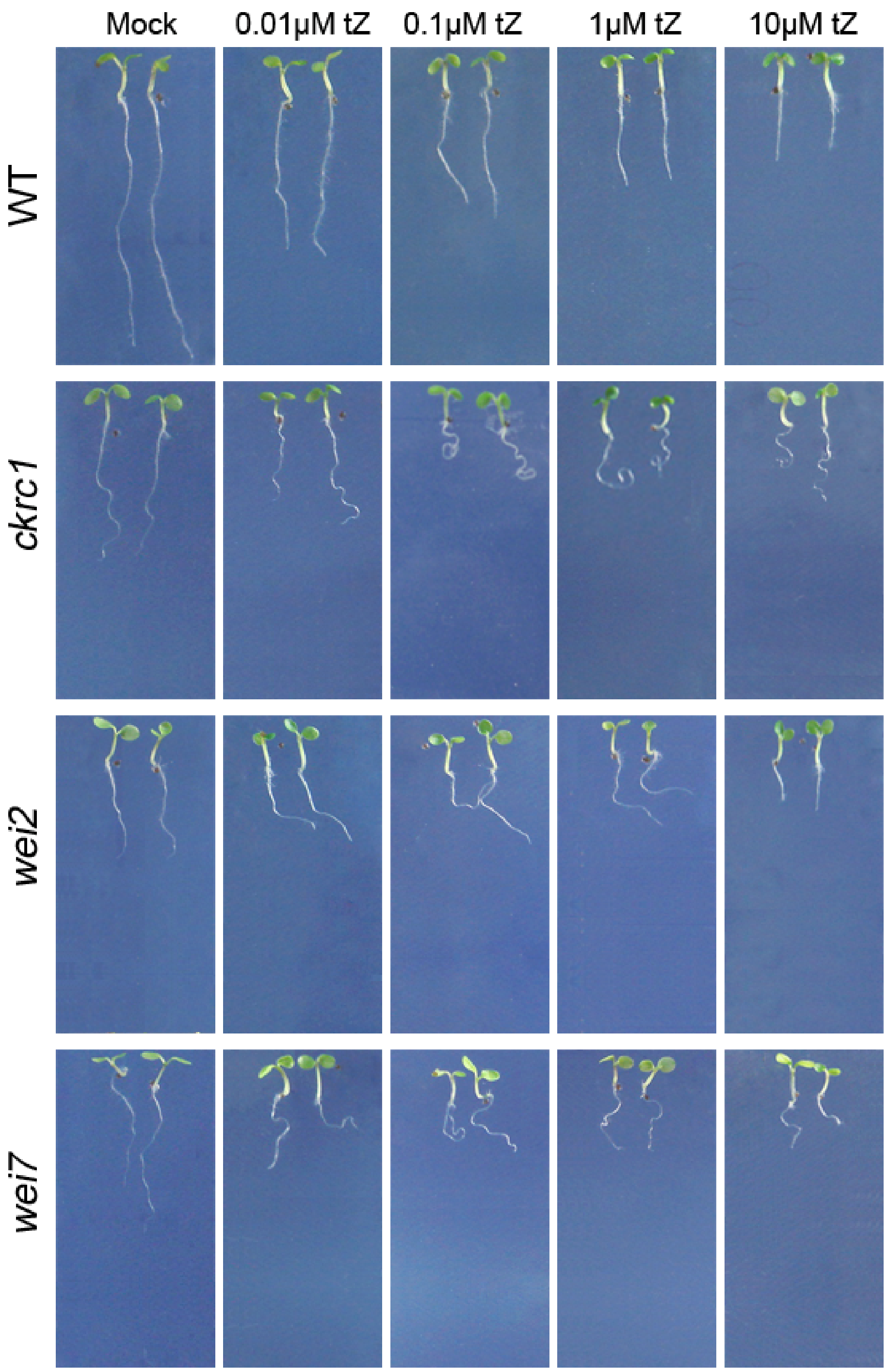

Supplementary Fig. S1 Root phenotypes of WT, *ckrc1-1*, *wei2* and *wei7* seedlings on MS plates with indicated cytokinin concentrations 7 DAG.

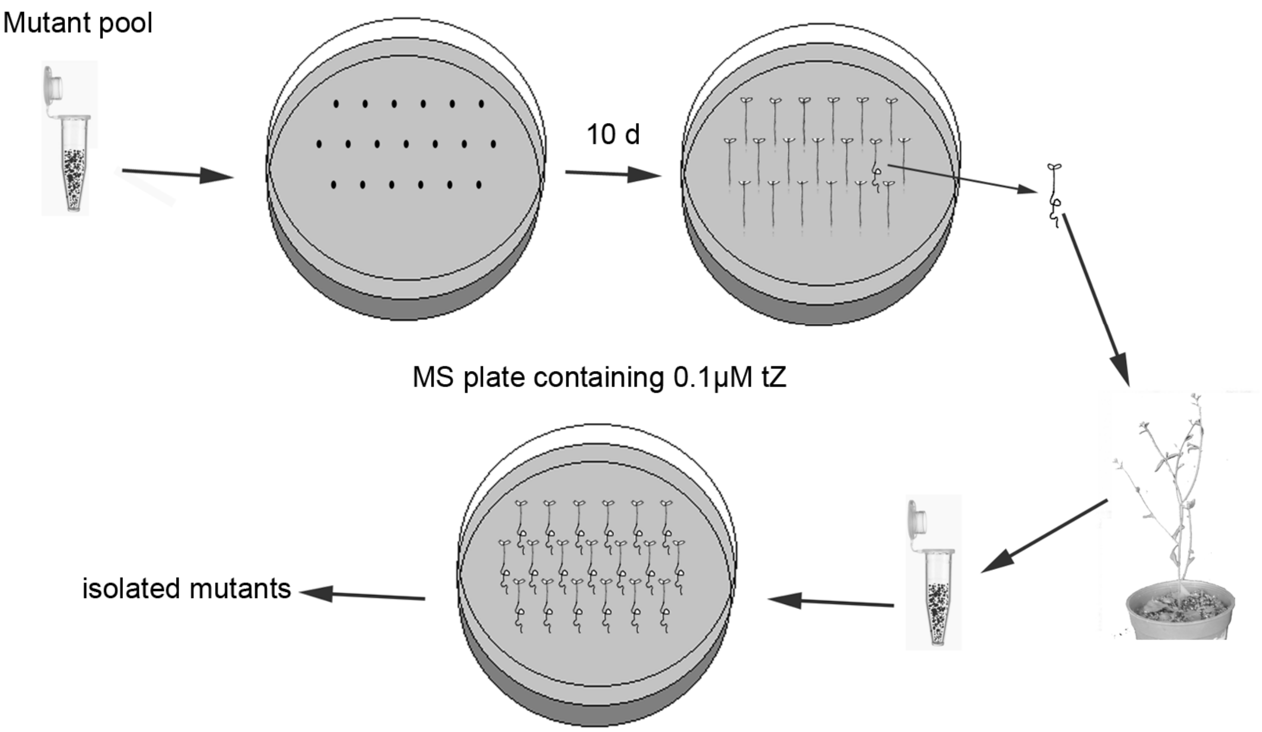

Supplementary Fig. S2 Schematic diagram of the two-round forward genetic screen for mutants using CK-induced root curling/waving as a phenotypic marker.

Supplementary Appendix S1 PCR primer sequences

QRT-ARR5-F: CCAGTCATCCCAGGCATAGAGT

QRT-ARR5-R: GATCGGAAGTTCATCGAGCGG

QRT-ARR15-F: GGAGTGTCGTCATCAAGGGAG

QRT-ARR15-R: GAGGTGGTGAAGCTGAAGAAGG

QRT-ACT8-F: TGTGACAATGGTACTGGAATGG

QRT-ACT8-R: TTGGATTGTGCTTCATCACC

QRT-IAA1F(2): CGGTTAGATCTCACTGGAGGCCAT

QRT-IAA1R(2): ATCTGCTCCTCCTCCTGCAAAAAC

QRT-IAA2F: AGGAAGAGTCTAGAGCAGGAGC

QRT-IAA2R: ACTGGATGTTGGTTGGTGATG

Supplementary Table S1 Summary information about the 53 mutants with curling (rc) or waving (rw) roots following tZ exposure obtained in the first large-scale screen.

Mutant	T-DNA vector	Dominant/ recessive(r)	Flanking sequence	Allele	Group
rc1	pROK2	r	AT2g39990	*pin2*	I
rc2	pROK2	r		*pin2*	I
rc3	pROK2	r	**AT2g38120 (*AUX1*)**	*aux1*	I
rc4	pROK2	r	**AT1g70560 (*CKRC1*)**	*ckrc1*	II
rc5	pROK2	r	**AT1g70560 (*CKRC1*)**	*ckrc1*	II
rc6	pROK2	r	AT1g01650; AT4g21903; **AT5g57090 (*PIN2*)**	*pin2*	I
rc7	pROK2	r	AT2g43140-AT2g43150; **AT2g38120 (*AUX1*)**	*aux1*	I
rc8	pROK2	r	AT1g53930-AT1g53935; **AT1g70560 (*CKRC1*)**	*ckrc1*	II
rc9	pROK2	r	**AT2g38120 (*AUX1*)**	*aux1*	I
rc10	pROK2	r	AT2g11740; AT1g53930-AT1g53935; AT2g47130-AT2g47140	*pin2*	I
rc11	pROK2	r	AT1g61940-AT1g61950	*aux1*	I
rc12	pSKI015	r		*aux1*	I
rc13	pSKI015	r	AT4g27745	*wei7*	II
rc14	pSKI015	r	AT4g27745	*ckrc1*	II
rc15	pSKI015	r	AT4g27745; **AT1g70560 (*CKRC1*)**	*ckrc1*	II
rc16	pSKI015	r	**AT1g70560 (*CKRC1*)**	*ckrc1*	II
rc17	pSKI015	r	AT5g04340-AT5g04347	*aux1*	I
rc18	PROK2	r	AT1g61940-AT1g61950	*aux1*	I
rc19	pSKI015	r	**AT1g70560 (*CKRC1*)**	*ckrc1*	II
rc20	pSKI015	r	**AT1g70560 (*CKRC1*)**	*ckrc1*	II
rc21	pSKI015	r	**AT2g38120 (*AUX1*)**	*aux1*	I
rc22	pSKI015	r	AT2g17390; **AT1g70560 (*CKRC1*)**	*ckrc1*	II
rc23	pSKI015	r	AT3g55210-AT3g55220; **AT2g38120 (*AUX1*)**	*aux1*	I
rc24	pSKI015	r	AT2g46950 (CYP709B2)	*wei2*	II
rc25	pSKI015	r	AT3g55210-AT3g55220	*aux1*	I
rc26*	**pSKI015**	**r**		***ckrc2****	**II**
rc27	pSKI015	r	AT4g01260-AT4g01265	*aux1*	I
rc28	pSKI015	r	AT1g71280-AT1g71290; AT5g48000-AT5g48010	*aux1*	I
rc29	pSKI015	r	**AT2g38120 (*AUX1*)**	*aux1*	I
rc30	pSKI015	r	**AT5g57090 (*PIN2*)**	*pin2*	I
rc31	pSKI015	r	AT2g14450-AT2g14455; AT2g17930	*aux1*	I
rc32	pSKI015	r	AT3g55210-AT3g55220; **AT2g38120 (*AUX1*)**	*aux1*	I
rc33	pSKI015	r	AT3g51860-AT3g51870	*aux1*	I
rc34	pSKI015	r		*pin2*	I
rw2*	**pSKI015**	**r**	AT5g25425-AT5g25430	***ckrw2****	**III**

rc36	pSKI015	r		*wei7*	II
rc37	pSKI015	r		*aux1*	I
rc38	pSKI015	r		*pin2*	I
rw1*	**pSKI015**	**r**	**AT5g49665 (*WAV3*);** AT4g18430; AT4g08090	***ckrw1****	**III**
rc40	pSKI015	r		*aux1*	I
rc41	pSKI015	r		*ckrc1*	II
rc42	pSKI015	r		*aux1*	I
rc43	pSKI015	r		*wei7*	II
rc44	pSKI015	r		*pin2*	I
rc45	pSKI015	r		*pin2*	I
rc46	pSKI015	r	AT1g15680-AT1g15690; AT1g15780; AT2g14450-AT2g14455	*pin2*	I
rc47	pSKI015	r	AT4g27745; AT1g71280-AT1g71290; AT1g15780	*wei7*	II
rc48	pSKI015	r	AT2g14450-AT2g14455; AT5g06610	*wei7*	II
rc49	pSKI015	r	AT2g14450-AT2g14455; AT1g68820-AT1g68825; **AT1G70560 (CKRC1)**	*ckrc1*	II
rc50	pSKI015	r		*wei2*	II
rc51	pSKI015	r	AT2g14450-AT2g14455	*aux1*	I
rc52	pSKI015	r	AT5g14910-AT5g14920; AT2g14450-AT2g14455; **AT1g70560 (CKRC1)**	*ckrc1*	II
rc53	pSKI015	r		*ckrc1*	II

Supplementary Table S2 Summary information about the 96 mutants with curling or waving roots following tZ exposure obtained in the subsequent large-scale screen.

Pools	Vector	Ecotype	No. of lines	No. seedlings screened	No. isolated mutants	No. confirmed known alleles	New rc/rw mutants	Group
CS 31400	pSKI015	C24	~9,700	~24,700	2			
CS 31402	pSKI015	C24	~4,000	~10,320	1	1 *aux1*		I
CS 21991	pSKI015	Col-7	~8,200	~108,810	6	3 *aux1*,<br>2 *pin2*		I<br>I
CS 21995	pSKI015	Col-7	~8,600	~48,860	1			
CS 23153	pSKI015	Col-7	~6,200	~63,900	16	1 *ckrc1*,<br>5 *pin2*,<br>1 *wei2*,	*ckrc3*(*ddw41*)	II<br>I<br>II
CS 31087	pD991	Col-6	~11,30<br>0	~113,060	16	2 *ckrc1*,<br>9 *aux1*,<br>1 *pin2*	*ckrc4*(*zb11*)<br>*crc1*(*le10*)	II<br>I<br>I
CS 22830	pD991-AP3	Ws-2	~37,80<br>0	~408,140	11	4 *aux1*,<br>1 *pin2*,		I<br>I

						1 *tir1*	*crc4*(*za212*)	I
							crc2(*lb2*)	I
							ckrc2-2(*lb72*)	II
							ckrc5(*lb113*)	II
CS 6502	3850:1003	Ws-2	~6,500	~71,580	4			
CS 84442	3850:1003	Ws-2	~4,000	~40,050	39	32 *pin2*, 1 *aux1*,	*crc3*(*c5*)	I
Total			~59,600	~632,830	96	64	8	
